# Risk of Early Mortality in Patients With Newly Diagnosed Multiple Myeloma: Erratum

**DOI:** 10.1097/01.md.0000480958.71776.2a

**Published:** 2016-02-12

In the article “Risk of Early Mortality in Patients With Newly Diagnosed Multiple Myeloma”,^1^ which appeared in Volume 94, Issue 50 of *Medicine*, some redundant values were originally presented in the abstract. The article has since been corrected online.
